# Supplementary material for: Mechanisms governing the pioneering and redistribution capabilities of the non-classical pioneer PU.1
Source: Nat Commun. 2020 Jan 21;11:402. doi: 10.1038/s41467-019-13960-2 (PMC6972792; doi:10.1038/s41467-019-13960-2)
Supplement: Supplementary file 7 — Source data [file 41467_2019_13960_MOESM7_ESM.zip › Source_Data/Figure2/Figure2F_MetascapeResults/Enrichment_GO/ColorByCluster.pdf]

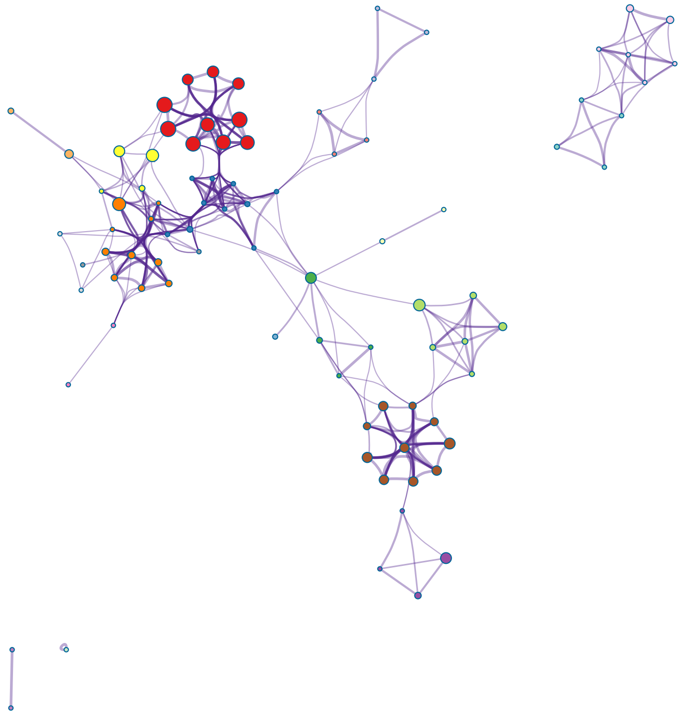

myeloid leukocyte activation
mast cell activation
leukocyte migration
small GTPase mediated signal transduction
B cell proliferation
adaptive immune response
cellular calcium ion homeostasis
response to hydroperoxide
inflammatory response to antigenic stimulus
Synthesis of Leukotrienes (LT) and Eoxins (EX)
Immunoregulatory interactions between a Lymphoid [illegible]
negative regulation of reactive oxygen species metab[illegible]
response to amphetamine
Cell surface interactions at the vascular wall
myeloid leukocyte differentiation
insulin secretion
acid secretion
regulation of protein deacetylation
Molecules associated with elastic fibres
cellular response to UV

created by
http://metascape.org
